# Comparative Evaluation of Calendula officinalis and Povidone-Iodine in Facial Wound Healing

**DOI:** 10.7759/cureus.110960

**Published:** 2026-06-16

**Authors:** Reshma Hammannavar, Harshal Patil, Uday D Berad, Ashlesha P Borse, Shruti S Joshi, Vaishnavi M Bhavsar

**Affiliations:** 1 Department of Oral and Maxillofacial Surgery, Jawahar Medical Foundation’s Annasaheb Chudaman Patil Memorial Dental College, Dhule, IND

**Keywords:** calendula officinalis, facial wounds, oral surgery, povidone iodine, wound healing

## Abstract

Introduction and aim: Facial wound healing is an important aspect of postoperative care in oral and maxillofacial surgery, because optimal healing is essential for both functional and esthetic outcomes. Topical agents with antimicrobial and anti-inflammatory properties are routinely used to enhance wound healing and to prevent postoperative complications. This study aimed to evaluate and compare the effectiveness of *Calendula officinalis* tincture and povidone-iodine ointment in the management of facial wounds. This study aimed to compare wound-healing outcomes between the two groups using the Early Wound Healing Score (EHS), evaluate the efficacy of *Calendula officinalis* tincture in promoting wound healing, assess the efficacy of povidone-iodine ointment in postoperative wound care, compare the incidence of wound infection or dehiscence between the groups, and evaluate patient satisfaction following topical wound management. The primary outcome of this study was the total Early Wound Healing Score (EHS) at day 14, while key secondary outcomes included the clinical signs of inflammation (CSI) score, wound infection rate, and patient satisfaction.

Materials and methods: This prospective observational clinical study included 64 participants with facial wounds who required postoperative wound care. Participants were divided into two observational groups based on the topical medication prescribed during routine clinical practice. Group A included 32 participants treated with *Calendula officinalis* tincture, while group B included 32 participants treated with povidone-iodine ointment. Wound healing was assessed using the Early Wound Healing Score (EHS), which included clinical signs of re-epithelialization, hemostasis, and inflammation at baseline and on days three, seven, and 14. Statistical analysis was performed using Mann-Whitney U test, Friedman test, and chi-square test. Statistical significance was set at p < 0.05.

Results: The mean age of participants was 31.6 ± 9.4 years in group A and 33.1 ± 10.2 years in group B. Baseline EHS scores were comparable between the groups (p > 0.05). A significant improvement in wound healing was observed in both groups throughout the follow-up period (p < 0.001). However, group A demonstrated significantly higher total EHS scores than group B from day three onwards. At day 14, the mean total EHS was 7.81 ± 0.61 in group A compared to 7.06 ± 0.87 in group B (p = 0.001). Group A also showed significantly better re-epithelialization and lower inflammation scores during follow-up evaluation.

Conclusion: *Calendula officinalis* tincture and povidone-iodine ointment promote satisfactory facial wound healing. However, *Calendula officinalis* tincture demonstrated superior healing outcomes with improved re-epithelialization and reduced inflammation, suggesting its potential as an effective topical agent for postoperative facial wound management.

## Introduction

Facial wounds are commonly encountered in oral and maxillofacial surgical practice and may arise following surgical procedures or traumatic injuries. Because the face has significant esthetic and functional importance, appropriate postoperative wound management is essential to achieve satisfactory healing with minimal complications and scarring [1]. Wound healing is a dynamic and complex biological process involving hemostasis, inflammation, proliferation, and remodeling, all of which must occur in a coordinated manner for optimal tissue repair [2]. Disruption at any of these stages may delay healing and increase the risk of infection, wound dehiscence, and poor cosmetic outcomes.

Prevention of wound infection remains one of the primary objectives of postoperative wound care. As facial wounds are frequently exposed to oral flora and environmental contaminants, the use of topical antimicrobial agents plays an important role in reducing microbial load and promoting favorable healing conditions [3]. Povidone-iodine is one of the most widely used antiseptic agents in clinical practice because of its broad-spectrum antimicrobial activity against bacteria, fungi, viruses, and protozoa [4]. It is also known for its biofilm-penetration ability, low microbial resistance, and cost-effectiveness, making it a preferred dressing agent for oral and maxillofacial surgery [5].

In recent years, increasing attention has been directed toward herbal and naturally derived agents that may enhance wound healing with fewer adverse effects. *Calendula officinalis*, commonly known as pot marigold, possesses anti-inflammatory, antimicrobial, antioxidant, and tissue-regenerative properties attributed to its bioactive compounds, such as flavonoids, triterpenoids, carotenoids, and phenolic acids [6]. Previous studies have demonstrated that *Calendula officinalis* promotes collagen synthesis, fibroblast proliferation, epithelialization, and angiogenesis, thereby accelerating wound healing and reducing inflammation and scar formation [7,8]. Owing to these properties, *Calendula officinalis* tincture has emerged as a promising alternative topical agent for wound management [8]. Although both povidone-iodine and *Calendula officinalis* are used in wound care, limited evidence is available to compare their effectiveness in facial wound healing following oral and maxillofacial surgical procedures.

## Materials and methods

Study design and setting

This study was designed as a prospective observational clinical study conducted in the Department of Oral and Maxillofacial Surgery at Jawahar Medical Foundation’s Annasaheb Chudaman Patil Memorial Dental College, Dhule, Maharashtra, India. This study was carried out over a period of one year (May 2023 to April 2024) after obtaining approval from the Institutional Ethics Committee (IEC no. EC/NEW/INST/2022/2959/2023/02D). Written informed consent was obtained from all the participants prior to their inclusion in the study.

Study population and sample size estimation

A total of 64 participants fulfilling the eligibility criteria were included in this study. The sample size was estimated using G*Power version 3.1 (Düsseldorf, Germany: Heinrich Heine University), based on previously published literature comparing wound-healing outcomes between *Calendula officinalis* and povidone-iodine preparations [9]. Considering a confidence level of 95% and study power of 80%, a minimum sample size of 32 participants per group was calculated to detect a clinically significant difference in wound-healing outcomes between the two treatment modalities. Therefore, 64 participants were included in this study.

Participants were not randomized. Instead, assignment to either* Calendula officinalis *tincture (group A) or povidone-iodine ointment (group B) was determined by routine clinical practice, based on the treating surgeon’s preference, wound characteristics (e.g., suspected contamination, presence of edema), and patient acceptance. To reduce selection bias, the following measures were taken: all eligible patients were enrolled consecutively. No specific wound characteristic was used as a rigid criterion for assignment; however, we recorded wound type (surgical versus traumatic), site, and baseline Early Wound Healing Score (EHS) to assess post hoc comparability between groups, thereby maintaining the observational nature of the study.

Inclusion and exclusion criteria

Patients aged 18-60 years with clean facial surgical wounds or traumatic facial wounds requiring postoperative wound dressing and follow-up care were included in this study. Only patients who were willing to participate, provided written informed consent, and were available for regular follow-up evaluation were considered eligible.

Exclusion criteria included uncontrolled systemic diseases affecting wound healing (such as uncontrolled diabetes mellitus or immunocompromised conditions), infected wounds at baseline, known allergy or hypersensitivity to *Calendula officinalis* or povidone-iodine, use of corticosteroids, chemotherapy, or immunosuppressive therapy, pregnancy or lactation, and chronic tobacco or alcohol abuse.

Clinical procedure

**Figure 1:**
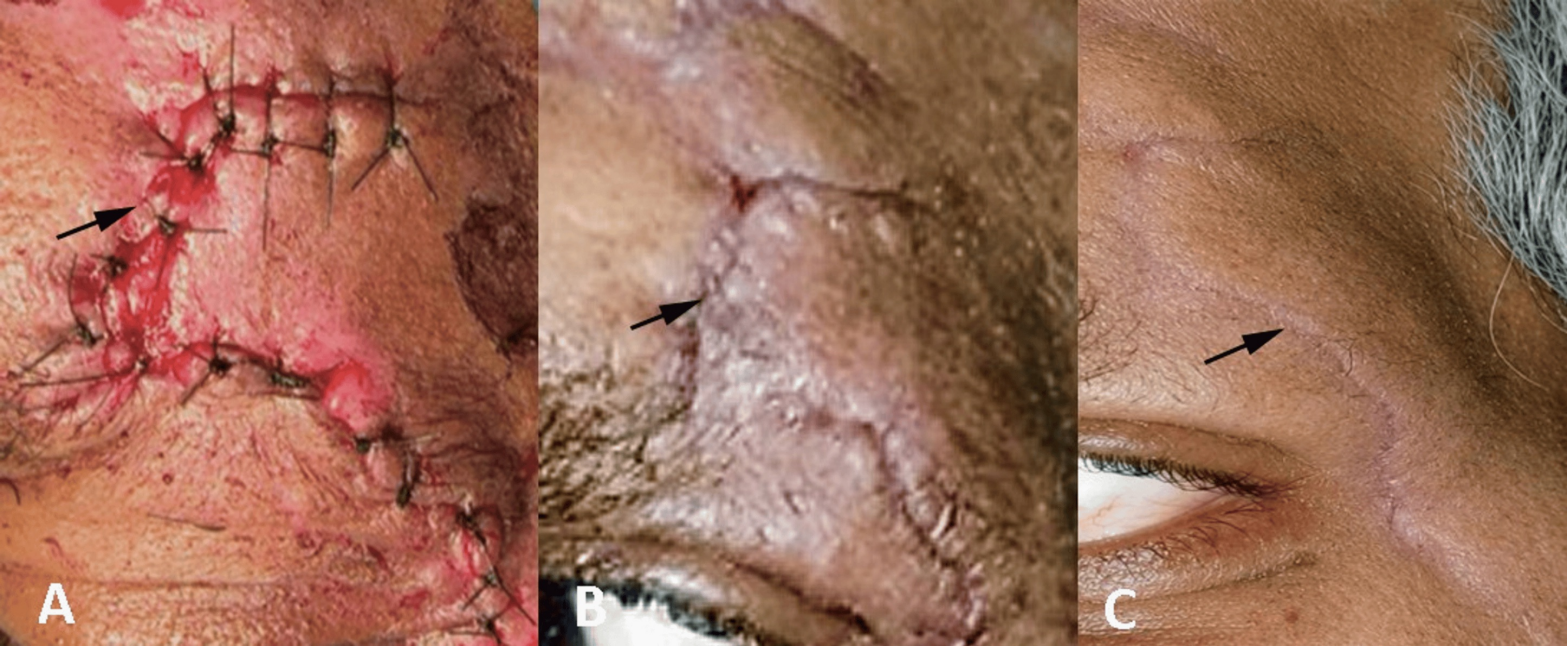
Clinical examination of wound healing in the Calendula officinalis tincture group at day three (A), day seven (B), and day 14 (C). Black arrow showing healing area.

Following completion of the surgical procedure and achievement of hemostasis, standard wound closure was performed using appropriate suturing techniques. The baseline clinical evaluation of the wound was recorded immediately after surgery. In group A, *Calendula officinalis* tincture (Ghaziabad, India: SBL Pvt. Ltd.) was applied topically over the wound area using sterile gauze or cotton applicators according to standard dilution protocols (Figures 1A-1C).

A commercially available *Calendula officinalis* mother tincture was diluted 1:10 with sterile normal saline (0.9% NaCl) immediately before each application to achieve a final concentration of 10% v/v. The dilution was prepared in a sterile 5 mL syringe. For each dressing, 0.5 mL of the diluted tincture was applied evenly over the wound surface using a sterile cotton applicator, covering the entire suture line and extending 2-3 mm beyond the wound margins. Excess solution was allowed to air-dry for 30 s before wound closure.

**Figure 2:**
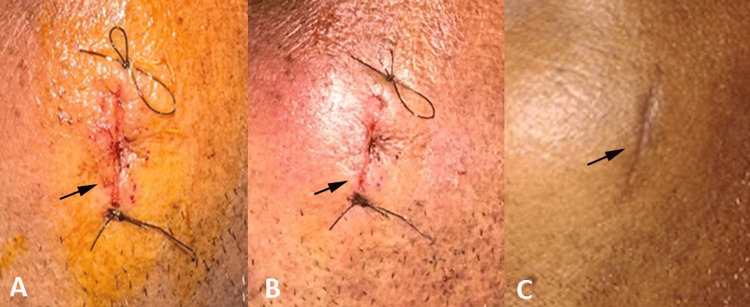
Clinical examination of wound healing in the povidone-iodine ointment group at day 3 (A), day 7 (B), and day 14 (C). Black arrow showing the healing area.

In group B, povidone-iodine ointment 5% w/w (New Delhi, India: Win-Medicare Pvt. Ltd.) was applied uniformly over the wound surface (Figures 2A-2C). A 5% w/w povidone-iodine ointment was applied as a thin layer (approximately 0.2-0.3 mm thickness) using a sterile spatula. The exact amount was not weighed, but visual standardization was ensured by using a standard 2‑cm length of ointment ribbon from a 15‑mm nozzle tube, corresponding to approximately 0.2 g per dressing.

Patients in both groups received identical postoperative instructions, oral medications when required, and routine wound care advice. Topical application was advised twice daily for a period of seven to 14 days, depending on wound characteristics and healing response.

Outcome assessment

All wound evaluations were performed by a single trained assessor who was blinded to group allocation and was not involved in the patient’s treatment or wound dressing application. The operator (treating surgeon) did not perform the final outcome assessments. Wound healing was assessed using the EHS proposed by Marini et al. [10]. The EHS comprised the following three clinical parameters: clinical signs of re-epithelialization (CSR), clinical signs of hemostasis (CSH), and clinical signs of inflammation (CSI). CSR was scored from 0 to 6, whereas CSH and CSI were scored from 0 to 2. The cumulative EHS ranged from 0 to 10, with higher scores indicating better wound healing. This scoring system is an open-access system distributed under the terms and conditions of the Creative Commons Attribution (CCBY) license (http://creativecommons.org/licenses/by/4.0/).

Intraobserver reliability was assessed by the single blinded examiner re‑scoring 20 randomly selected wounds (10 per group) at two timepoints, 48 h apart. The intraclass correlation coefficient (ICC) for total EHS was 0.96 (95% CI: 0.91-0.98), indicating excellent intraobserver agreement. All sub‑scores (CSR, CSH, CSI) showed ICCs ≥ 0.90.

Clinical evaluation was performed at baseline (day 0) and on postoperative days three, seven, and 14. The primary outcome measure was the comparative assessment of wound healing between the two observational groups using the EHS parameters. Secondary outcome measures included the incidence of wound infection, wound dehiscence, and patient satisfaction with postoperative wound healing.

Statistical analysis

The collected data were analyzed using Statistical Package for the Social Sciences (SPSS) software version 26.0 (Armonk, NY: IBM Corp.). Descriptive statistics are expressed as mean ± standard deviation for continuous variables and frequency with percentage for categorical variables. The normality of EHS scores was assessed using the Shapiro-Wilk test, which demonstrated a non-normal distribution of the data. Intergroup comparisons of EHS parameters were performed using the Mann-Whitney U test, while intragroup comparisons across different follow-up intervals were analyzed using the Friedman repeated-measures test. Categorical variables were compared using the chi-square test or Fisher’s exact test, as appropriate. Statistical significance was set at p < 0.05.

## Results

A total of 64 participants were included, with 32 in each group. The mean age in group A was 31.6 ± 9.4 years, while in group B it was 33.1 ± 10.2 years, with no statistically significant difference between the groups (p = 0.501). Group A included 18 males (56.3%) and 14 females (43.8%), whereas group B included 20 males (62.5%) and 12 females (37.5%). Surgical wounds were observed in 22 participants (68.8%) in group A and 24 participants (75.0%) in group B. Controlled diabetes mellitus was present in three participants (9.4%) in group A and four participants (12.5%) in group B. No statistically significant difference was observed between the groups for any demographic or clinical variable (p > 0.05) (Table 1).

**Table 1:** Demographic and clinical profile of study participants. Data were presented as mean ± standard deviation and frequency with percentage; independent samples t-test and chi-square test were used for comparison; p < 0.05 was considered statistically significant. t: independent samples t-test statistic, χ²: chi-square statistic

Characteristics	Category	Group A (*Calendula officinalis*), n = 32	Group B (povidone-iodine), n = 32	Test value	p-Value
Age (years)	Mean ± SD	31.6 ± 9.4	33.1 ± 10.2	t = -0.677	0.501
Sex, n (%)	Male	18 (56.3)	20 (62.5)	χ² = 0.254	0.614
	Female	14 (43.8)	12 (37.5)		
Wound type, n (%)	Surgical	22 (68.8)	24 (75.0)	χ² = 0.327	0.567
	Traumatic	10 (31.3)	8 (25.0)		
Site of wound, n (%)	Mandibular region	14 (43.8)	13 (40.6)	χ² = 0.578	0.749
	Maxillary region	10 (31.3)	11 (34.4)		
	Zygomatic/cheek	8 (25.0)	8 (25.0)		
History of diabetes, n (%)	Controlled DM	3 (9.4)	4 (12.5)	χ² = 0.182	0.670
	None	29 (90.6)	28 (87.5)		

The mean total EHS at baseline was 3.91 ± 1.02 in group A and 4.03 ± 0.98 in group B, with no statistically significant difference between the groups (p = 0.831). Similarly, the clinical signs of re-epithelialization (CSR), clinical signs of hemostasis (CSH), and clinical signs of inflammation (CSI) scores were comparable between the groups at baseline (p > 0.05), indicating a similar initial wound-healing status (Table 2).

**Table 2:** Comparison of baseline EHS parameters between groups. Data were presented as mean ± standard deviation; Mann-Whitney U test was used for intergroup comparison; p < 0.05 was considered statistically significant. EHS: Early Wound Healing Score; CSR: clinical signs of re-epithelialization; CSH: clinical signs of hemostasis; CSI: clinical signs of inflammation

EHS parameter	Group A (*Calendula officinalis*), mean ± SD	Group B (povidone-iodine), mean ± SD	Mean difference	Mann-Whitney U stats	p-Value
CSR (0-6)	1.88 ± 0.74	1.94 ± 0.70	0.06	488	0.812
CSH (0-2)	1.16 ± 0.44	1.19 ± 0.47	0.03	501	0.926
CSI (0-2)	0.88 ± 0.34	0.91 ± 0.37	0.03	494	0.871
Total EHS (0-10)	3.91 ± 1.02	4.03 ± 0.98	0.12	489	0.831

In group A, the total EHS improved significantly from 3.91 ± 1.02 at baseline to 7.81 ± 0.61 at day 14 (p < 0.001). Similarly, group B demonstrated a significant increase in total EHS from 4.03 ± 0.98 at baseline to 7.06 ± 0.87 at day 14 (p < 0.001). Significant improvements in CSR, CSH, and CSI scores were observed in both groups across all follow-up intervals (p < 0.001) (Table 3).

**Table 3:** Intragroup comparison of early wound healing score parameters across follow-up intervals. *Indicates statistically significant difference. Data were presented as mean ± standard deviation; Friedman repeated-measures test was used for intragroup comparison across follow-up intervals; p < 0.05 was considered statistically significant. χ²: Friedman test statistic; EHS: Early Wound Healing Score; CSR: clinical signs of re-epithelialization; CSH: clinical signs of hemostasis; CSI: clinical signs of inflammation

Outcome measure	Baseline day 0	Day 3	Day 7	Day 14	Test value (χ²)	p-Value
Group A - *Calendula officinalis *(n = 32)						
CSR	1.88 ± 0.74	3.25 ± 0.87	4.69 ± 0.72	5.78 ± 0.43	78.4	<0.001*
CSH	1.16 ± 0.44	1.53 ± 0.51	1.81 ± 0.40	1.94 ± 0.24	44.1	<0.001*
CSI	0.88 ± 0.34	0.53 ± 0.31	0.28 ± 0.22	0.09 ± 0.10	62.7	<0.001*
Total EHS	3.91 ± 1.02	5.31 ± 1.14	6.78 ± 0.98	7.81 ± 0.61	88.3	<0.001*
Group B - povidone-iodine (n = 32)						
CSR	1.94 ± 0.70	2.84 ± 0.79	3.97 ± 0.84	5.00 ± 0.66	71.9	<0.001*
CSH	1.19 ± 0.47	1.44 ± 0.50	1.66 ± 0.48	1.81 ± 0.40	38.2	<0.001*
CSI	0.91 ± 0.37	0.72 ± 0.33	0.50 ± 0.28	0.25 ± 0.18	52.3	<0.001*
Total EHS	4.03 ± 0.98	4.97 ± 1.07	6.13 ± 1.09	7.06 ± 0.87	76.4	<0.001*

At baseline, no statistically significant differences were observed between the groups for any of the EHS parameters (p > 0.05). However, from day three onward, group A demonstrated significantly better CSR and total EHS scores, along with lower CSI scores, compared with group B. On day 14, the mean total EHS was significantly higher in group A (7.81 ± 0.61) than in group B (7.06 ± 0.87) (p = 0.001) (Table 4).

**Table 4:** Intergroup comparison of early wound healing score parameters at different follow-up intervals. *Indicates statistically significant difference. Data were presented as mean ± standard deviation; Mann-Whitney U test was used for intergroup comparison at each follow-up interval; p < 0.05 was considered statistically significant. EHS: Early Wound Healing Score; CSR: clinical signs of re-epithelialization; CSH: clinical signs of hemostasis; CSI: clinical signs of inflammation

Time point	EHS parameter	Group A (mean ± SD)	Group B (mean ± SD)	Mean difference	Mann-Whitney U stats	p-Value
Baseline (day 0)	CSR	1.88 ± 0.74	1.94 ± 0.70	-0.06	488	0.812
	CSH	1.16 ± 0.44	1.19 ± 0.47	-0.03	501	0.926
	CSI	0.88 ± 0.34	0.91 ± 0.37	-0.03	494	0.871
	Total EHS	3.91 ± 1.02	4.03 ± 0.98	-0.12	489	0.831
Day 3	CSR	3.25 ± 0.87	2.84 ± 0.79	+0.41	352	0.031*
	CSH	1.53 ± 0.51	1.44 ± 0.50	+0.09	460	0.389
	CSI	0.53 ± 0.31	0.72 ± 0.33	-0.19	383	0.044*
	Total EHS	5.31 ± 1.14	4.97 ± 1.07	+0.34	381	0.042*
Day 7	CSR	4.69 ± 0.72	3.97 ± 0.84	+0.72	284	0.003*
	CSH	1.81 ± 0.40	1.66 ± 0.48	+0.15	418	0.098
	CSI	0.28 ± 0.22	0.50 ± 0.28	-0.22	310	0.008*
	Total EHS	6.78 ± 0.98	6.13 ± 1.09	+0.65	297	0.005*
Day 14	CSR	5.78 ± 0.43	5.00 ± 0.66	+0.78	248	<0.001*
	CSH	1.94 ± 0.24	1.81 ± 0.40	+0.13	412	0.112
	CSI	0.09 ± 0.10	0.25 ± 0.18	-0.16	274	<0.001*
	Total EHS	7.81 ± 0.61	7.06 ± 0.87	+0.75	256	0.001*

Significant differences were observed between the baseline and all subsequent follow-up intervals for the total EHS scores within group A (p < 0.001). Progressive improvement in wound healing scores was noted from day three to day 14, indicating continuous improvement in healing with *Calendula officinalis* tincture application (Table 5).

**Table 5:** Wound healing category at day 14 between the study groups. Data were presented as frequency with percentage; chi-square test was used for intergroup comparison; p < 0.05 was considered statistically significant. EHS: Early Wound Healing Score; χ² = chi-square statistic

Wound healing category (at day 14)	Group A (*Calendula officinalis*), n = 32 (100%)	Group B (povidone-iodine), n = 32 (100%)	Chi-square (χ²)	p-Value
Excellent (EHS 8-10), n (%)	20 (62.5%)	12 (37.5%)	χ² = 7.14	0.007*
Good (EHS 6-7), n (%)	9 (28.1%)	14 (43.8%)		
Fair (EHS 4-5), n (%)	3 (9.4%)	5 (15.6%)		
Poor (EHS < 4), n (%)	0 (0.0%)	1 (3.1%)		

Significant improvement in the total EHS scores was observed between the baseline and all subsequent follow-up intervals within group B (p < 0.001). Although wound healing improved progressively over time in group B, the degree of improvement was less pronounced than that observed in group A (Table 6).

**Table 6:** Secondary outcomes - wound complications and patient satisfaction. *P < 0.05 was statistically significant. Fisher's exact test was used for cell counts < 5; chi-square for larger cell counts; independent t-test for satisfaction scores. Group A showed significantly fewer overall complications (p = 0.048) and higher satisfaction at day seven and day 14. VAS: visual analog scale

Secondary outcome	Group A (*Calendula officinalis*)	Group B (povidone-iodine)	Test value	p-Value
Incidence of wound complications, n (%)				
Wound infection	1 (3.1)	4 (12.5)	Fisher's p	0.168
Wound dehiscence	0 (0.0)	2 (6.3)	Fisher's p	0.492
Delayed healing	2 (6.3)	5 (15.6)	χ² = 1.64	0.200
Any complication	3 (9.4)	9 (28.1)	χ² = 3.92	0.048*
Patient satisfaction score (VAS 0-10), mean ± SD				
Day 3	6.72 ± 1.04	6.28 ± 1.11	t = 1.753	0.084
Day 7	7.84 ± 0.92	7.22 ± 1.03	t = 2.706	0.008*
Day 14	8.56 ± 0.75	7.94 ± 0.98	t = 3.046	0.003*

## Discussion

Successful postoperative wound healing is an essential component of oral and maxillofacial surgical care, particularly for facial wounds, where both esthetic and functional outcomes are of considerable importance [2]. The present prospective observational clinical study evaluated and compared the effects of *Calendula officinalis* tincture and povidone-iodine ointment on facial wound healing following minor oral and maxillofacial surgical procedures. The findings of this study demonstrated that both topical agents promoted progressive wound healing; however, *Calendula officinalis* showed superior healing outcomes compared with povidone-iodine ointment at different follow-up intervals.

The baseline demographic and clinical characteristics of the participants were statistically comparable between the two groups, indicating homogeneity of the study population. No significant differences were observed with respect to age, sex distribution, wound type, wound site, or history of controlled diabetes mellitus. This ensured that the differences observed during follow-up were primarily attributable to the topical agents used rather than demographic confounding factors.

In the present study, significant improvements in all the EHS parameters were observed in both groups throughout the follow-up period. The total EHS scores increased progressively from baseline to day 14 in both groups, indicating satisfactory healing with *Calendula officinalis* tincture and povidone-iodine ointment. These findings are consistent with those of Vermeulen et al., who reported that povidone-iodine remains an effective antiseptic agent without adversely affecting wound healing [11]. Bigliardi et al. emphasized the broad antimicrobial activity and favorable wound-healing properties of povidone-iodine in acute and chronic wounds [12].

However, intergroup comparisons demonstrated significantly superior healing outcomes in the *Calendula officinalis* group from day three onwards. Participants treated with* Calendula officinalis* tincture showed significantly higher CSR and total EHS scores, along with lower CSI scores, compared with the povidone-iodine group. These findings suggest that *Calendula officinalis* may facilitate faster tissue regeneration and reduce the inflammatory response more effectively than povidone-iodine [7,8].

The enhanced wound healing observed with *Calendula officinalis *may be attributed to its rich phytochemical composition, including flavonoids, triterpenoids, carotenoids, and phenolic compounds, which possess anti-inflammatory, antioxidant, antimicrobial, and collagen-stimulating properties [8]. Previous experimental and clinical studies have demonstrated that *Calendula officinalis* promotes fibroblast proliferation, angiogenesis, collagen synthesis, and epithelialization, thereby accelerating wound healing [13]. Fronza et al. reported that *Calendula officinalis* tincture significantly stimulated fibroblast proliferation and migration through PI3K-dependent pathways, thereby contributing to improved tissue regeneration [14]. Similarly, Leach reported that topical calendula preparations improve epithelialization and reduce inflammation in acute and chronic wounds [15].

The present findings are also in agreement with those of Sharma et al., who observed better wound contraction, reduced inflammation, and faster healing in wounds treated with *Calendula officinalis* than in those treated with povidone-iodine [9]. In their systematic review, Givol et al. further highlighted the beneficial role of *Calendula officinalis* in accelerating the resolution of inflammation and granulation tissue formation [8]. Additionally, Rezai et al. demonstrated improved collagen organization and tensile strength with calendula application, supporting its role in enhancing tissue repair [16].

Although povidone-iodine demonstrated satisfactory wound-healing outcomes, its comparatively lower healing scores may be related to mild tissue irritation and cytotoxicity associated with prolonged use. Previous studies have suggested that while povidone-iodine effectively reduces microbial contamination, excessive or prolonged application may interfere with fibroblast activity and epithelial cell proliferation [17]. Nevertheless, its broad-spectrum antimicrobial action and affordability make it a widely accepted topical antiseptic for clinical practice.

The reduction in the inflammatory scores observed in the calendula group is clinically important because excessive inflammation may delay healing and contribute to scar formation. *Calendula officinalis *possesses natural anti-inflammatory properties that may help minimize tissue edema, erythema, and discomfort during postoperative healing [18]. Furthermore, the better patient satisfaction observed clinically in the *Calendula officinalis* group may be associated with reduced irritation and enhanced comfort during wound healing [8].

The findings of the present study have important clinical implications for oral and maxillofacial surgeries. Therefore, *Calendula officinalis *tincture may serve as a safe, economical, and effective herbal alternative to conventional antiseptic agents in postoperative facial wound management. Its natural origin, favorable healing response, and minimal adverse effects make it a promising adjunct to routine wound-care protocols.

However, certain limitations of this study should be considered when interpreting these results. The study had a relatively small sample size and a short follow-up duration. This observational design may also have introduced operator preference bias in the selection of topical agents. In addition, microbiological and histological evaluations of the wound healing were not performed. A further limitation is potential selection bias, as treatment assignment was clinician‑driven rather than randomized. Unreported wound characteristics (e.g., length, depth, contamination level) may have influenced both agent choice and healing outcomes, confounding the observed association between Calendula and improved scores. These factors warrant measurement in future trials. Therefore, further multicenter studies with larger sample sizes, longer follow-up periods, and advanced wound assessment parameters are recommended to validate these findings and to establish standardized clinical guidelines for the use of *Calendula officinalis* in facial wound healing.

## Conclusions

Both *Calendula officinalis* tincture and povidone-iodine ointment demonstrated significant improvements in facial wound healing following minor oral and maxillofacial surgical procedures. However, the *Calendula officinalis* tincture showed comparatively superior wound-healing outcomes, with better re-epithelialization, reduced inflammation, and higher overall EHS scores during the follow-up evaluation. These findings suggest that *Calendula officinalis* may be an effective and safe alternative topical agent for the management of postoperative facial wounds. Further large-scale longitudinal studies are recommended to validate the clinical efficacy and establish standardized therapeutic protocols.
